# Quantitative analysis of spontaneous sociality in children’s group behavior during nursery activity

**DOI:** 10.1371/journal.pone.0246041

**Published:** 2021-02-02

**Authors:** Jun Ichikawa, Keisuke Fujii, Takayuki Nagai, Takashi Omori, Natsuki Oka

**Affiliations:** 1 Faculty of Engineering, Kanagawa University, Yokohama, Kanagawa, Japan; 2 Graduate School of Informatics, Nagoya University, Nagoya, Aichi, Japan; 3 Graduate School of Engineering Science, Osaka University, Toyonaka, Osaka, Japan; 4 College of Engineering, Tamagawa University, Machida, Tokyo, Japan; 5 Faculty of Information and Human Sciences, Kyoto Institute of Technology, Kyoto, Kyoto, Japan; Institute of Physiology and Basic Medicine, RUSSIAN FEDERATION

## Abstract

Sociality is the tendency to spontaneously interact with others to establish and maintain relationships. Some approaches, including questionnaires, tests, controlled experiments, and qualitative field research, cannot capture complex social interactions, such as in children during nursery activities, because of problems with ecological validity and the labor cost of analysis. Here, we introduced a new methodology for the quantitative analysis of spontaneous social movement and investigated children’s group behavior using position data. We periodically visited a nursery and recorded videos of eurhythmics, in which children move in tune with music, in different classes. The results revealed that children in the six-year-old class approached others in a short period of time (within one second) and established group behavior like that in a game of tag. It can be interpreted that such social behavior may include actions related to the cognition of anticipating others’ behaviors in a complex situation. Although only a small amount of data could be acquired, this study suggests one of the characteristics of social behaviors in the classroom considering an ecological approach.

## Introduction

### Development of spontaneous sociality

Sociality is the tendency to spontaneously interact with others to establish and maintain relationships, according to the study of social interaction [1–3]. It is a foundation for coordination, joint action, and prosocial behavior. Humans show advanced and complex interactions with others through the cognition of estimating others’ intentions and anticipating others’ behaviors based on the sociality.

Developmental psychology and cognitive science studies have indicated that children develop the ability to estimate various internal states of others, including anticipating others’ behaviors, with increase of age (e.g. [4–6]). Understanding of simple others’ emotions, desires, and perceptions develops around two years old; however, understanding of others’ simple knowledge and beliefs does not develop at this stage [6]. It starts to develop from three or four years old and stabilizes by five years of age [5–8]. In addition, understanding of the intricate false beliefs of others concerning recursive relationships between two or more individuals develops around six years of age [9,10]. These previous studies have suggested that, although there are some cultural differences, estimating the internal states of others in more complex situations develops in stages by around six years of age. We can interpret that perceptually-guided estimation based on sociality may develop before six years old [11]. Many previous joint-action studies including two- and three-year-old children have discussed the importance of perceptually-guided anticipation of others’ behaviors in social interaction (e.g. [11–13]). Meanwhile, according to the findings of the previous studies [9,10], cognitively-guided estimation of such intricate beliefs may develop when children are six years old and the latter behaviors may be more social.

The motivation in this study is to quantitatively analyze the social behaviors of children around six years of age during nursery activity. We discuss children’s social interactions during a daily spontaneous playtime. Around six years of age, social behaviors related to the cognition of anticipating others’ behaviors in more complex situations may be observed. This study focuses on how engagements with others differ between age groups. These differences may capture the cognitive development of anticipating others’ behaviors based on spontaneous sociality.

### Approaches investigating sociality in children

There are four research approaches to assessing sociality in children. The first is a questionnaire. The advantage of this approach is scalability to dozens or hundreds of participants. Some studies of social interactions use questionnaires to examine children and their parents (e.g., [14,15]). The second approach is a test that is simple and easy for children to complete and this advantage is high reliability and validity because many previous studies have used them. Children complete a false-belief task, which is a test to investigate the age at which children can understand that others’ beliefs are sometimes different from their own and false (e.g., [4,7–10]). These tests indicate the estimation of the internal states of others. The third approach is a controlled experiment, which can avoid confounding because experimenters control the conditions. These are a standard psychological method used to investigate social interactions (e.g., [16,17]). However, regarding the research into the sociality of children, the three approaches sometimes have problems with ecological validity [18]. The problems related to ecological validity are that experimental environment differs from real life, and researchers sometimes cannot obtain participants’ true responses because researchers’ arbitrary operations intervene and participants’ behaviors are controlled to investigate the influences of specific factors [19]. While studying social interactions during nursery activities, some answers on a questionnaire may be biased, and researchers cannot estimate the extent to which behaviors depicted by the answers are implemented in a real-life scenario. The testing method has a similar problem. A controlled experiment involves the additional concern of whether behaviors required for participants are similar to those in real life.

The fourth approach is qualitative field research, which is based on observation and description of social interactions in a nursery or preschool [20–22]. Holding hands with others is a simple and spontaneous social behavior frequently observed in a nursery or preschool. A qualitative approach can solve problems with ecological validity and enables researchers to analyze details of behaviors in real life. However, it depends on the observers’ preferences for which points they pay attention to. Researchers monitoring and qualitatively analyzing many children at the same time would require too much labor cost [23,24]. Relationships with others and interpersonal attitudes are reflected by the inter-distance [25–27]. As a result, spontaneous diffusion and aggregation of children may indicate complex group behaviors based on sociality. However, it would be too difficult to define and analyze diffusion and aggregation through descriptions because such group behavior is established through momentary interactions with others based on relationships with others and interpersonal attitudes.

Therefore, it may be difficult to use the four approaches mentioned above to discuss the mechanism of complex group behavior of children during nursery activities. Quantitative analysis of group behavior is needed. Our methodology follows an ecological approach [18,19] and has a motivation of discussing children’s natural social interactions as childminders in the nursery actually observe them.

### Our approach

In the previous sections, we explained the findings of sociality in children and the approaches to assessing it in developmental psychology and cognitive science studies. According to these disadvantages, this study introduced a new research methodology for the quantitative analysis of spontaneous social movement in a nursery, which is a naturalistic environment. We posit that the cognitive development based on the sociality may be reflected by group behavior of six-year-old children, and analyzed children’s group behavior using position data. Recently, group behaviors have been analyzed in many academic fields, such as animal group movements in biology (e.g., [28–30]) and teamwork in sports science (e.g., [31–33]). These disciplines use the position data of individuals to calculate distances between individuals or the orientation vector of each individual. Not only recording positions in the field but also a simulation has been conducted (e.g., [29,30,33]). Most previous studies on animal group movements and human sports behaviors, including simulation studies, suppose that individuals move based on simple rules and regulations. However, children do not always move based on rules but often act on impulses during activities. It would, therefore, be more difficult for researchers to analyze the group behavior of children than those of animals or even sports players. This study presents a challenge for scientific understanding of group behaviors by a new research methodology, instead of other approaches of questionnaires, tests, controlled experiments, and qualitative field research. Our analysis was also used in a previous study sports science [34]. Group behavior in sports shows characteristic interactions between players over a brief period. Such interactions determine the winners of games within seconds. We posit that children’s group behavior over a brief period would show important development because childminders in the nursery often estimate children’s various internal states through experience and momentary observations.

We periodically visited the nursery and recorded videos of children’s activities in different grades. This study investigated children’s group behavior through the quantitative analysis of spontaneous social movement using position data. Few studies have quantitatively analyzed the sociality observed in the field. Nakamae et al. [35] analyzed where, with whom, and with what a child was playing using Bluetooth Low Energy (BLE) and an accelerometer. However, this was mainly done to avoid danger, and the mechanism of group behavior of how the child played with others was not fully discussed.

A body expression is formed in the moment through the interaction between one’s attitude and surrounding elements [36]. In addition, physical interaction is a basic means of constructing relationships with others [37]. Further, relationships with others are represented in physical interaction [38]; for example, a positive interpersonal attitude is expressed as approaching behavior [27]. Hence, we assumed that spontaneous sociality is represented by body movement. Hepach et al. quantitatively analyzed children’s change in posture and linked the characteristics with fulfillment after prosocial behavior [39]. Our work investigated embodied sociality, which is more interactive than gesture and posture. Needham and Libertus [40] note that embodiment is important for understanding development of children in actual situations, and works related to this subject are expected to be invaluable for academic researchers. Fantasia et al. also point out that an ecological approach is critical to understanding sociality and development more deeply [41]. This study analyzed spontaneous social movement during nursery activities, which indicates complex interactions in a classroom, considering an ecological approach. We found the possibility that the differences in children’s group behavior between age groups might be related to the findings of cognitive development in developmental psychology and cognitive science. Quantitative analysis and visualization of children’s natural group behavior during nursery activities would provide meaningful information of social relationships between children for childminders.

We focused on children’s group behavior during eurhythmics, in which they move in tune with music. Recently, educational institutions have paid attention to eurhythmics to develop sociality. The rhythm of music plays a role in mediating physical interaction with others [42,43]. In addition, music promotes social behaviors [44]. Eurhythmics are an appropriate environment for this study.

Here, our previous study serves as a preliminary investigation to implement our approach. In one project, we annotated videos of spontaneous social behaviors of each child with other children during running activities, which was the simplest action in eurhythmics (see details below studied activities in the Methods section) [45]. Specific haptic behaviors, which indicated spontaneous participation in group activities and involvements with others to establish good relationships, were annotated using ELAN software (https://tla.mpi.nl/tools/tla-tools/elan/), which loads a video and allows a user to perform manual annotations. Representative examples include holding hands with others and hugging. The important results confirmed that the frequency of such social behaviors per minute in the six-year-old class was higher than that in the five-year-old class. Behaviors in response to others or following social norms emerge with increase of age [46]. In addition, more social communication during activities at preschool unfolds at six years old [22,47]. Furthermore, estimating the internal states of others in more complex situations develops in stages by around six years of age [9,10]. Our preliminary investigation suggests that the group behavior of the six-year-old class might be more social than that of the five-year-old class because of the cognitive development of anticipating others’ behaviors.

### Hypothesizing children’s group behavior

Based on the previous sections, we think that analyzing behaviors of children around six years of age is important because, in this period, the cognition of anticipating others’ behaviors in complex situations develops and their behaviors may be more social. In addition, applying quantitative analysis used in studies of animal group movements and teamwork in sports is needed because it is difficult for our previous study mentioned above [45] to objectively explore the mechanism of group behavior based on spontaneous sociality through overall activities. This study quantitatively analyzed children’s group behavior using position data. Our previous study [45] was conducted to hypothesize children’s group behavior as a preliminary investigation. The findings provided necessary information to explain a basis for hypothesizing and we hypothesized group behavior related to the sociality in six-year-old children. In the running activity of eurhythmics of the six-year-old class, we predicted that three types of spontaneous social movements might emerge in the children’s group behavior. The first two are: #1) being close enough to touch each child and #2) running in the direction of another child to touch him/her. We expected that the frequencies of these actions in the six-year-old class would be higher than those in the five-year-old class. Furthermore, we watched the videos and confirmed that children in both classes ran around during the activities. However, it was also confirmed that, for the six-year-old class, the rotation movement of children was broken because they spontaneously communicated with others by touching. This study also hypothesized #3) running with a broken rotation movement to touch others. The frequency of strong rotation movements emerging in the six-year-old class would be lower than that in the five-year-old class.

We predicted age as a factor from which the three characteristics of the children’s group behavior would clearly emerge according to both the results of video annotations and the findings of previous studies related to sociality [9,10,22,47]. We expected that such behaviors of six-year-old children might be more social than those of five-year-old children because of the cognitive development of anticipating others’ behaviors in more complex situations. Next, the hypotheses were validated by analyzing group behavior in each class using position data.

## Methods

### Participants

This study investigated one of the activities of eurhythmics described below in two classes, five- and six-year-old classes. We expected that spontaneous social behaviors related to the cognition of anticipating others’ behaviors in complex situations might clearly emerge around six years of age. To further clarify this emergence, this study also observed the eurhythmics of the five-year-old class just before the six-year-old class.

The five- and six-year-old classes were composed of 13 children (boys: 6; girls: 7) and 13 children (boys: 5; girls: 8), respectively. For the former, we periodically observed and analyzed the group behaviors of the same children four times; we could examine the behaviors only once for the latter considering the nursery schedule and venue usage. The frequency of observation in the six-year-old class was lower than that in the five-year-old class because, in the six-year-old class, one of the activities of eurhythmics, which was modeled as an object of analysis, was not conducted, and children graduated from the nursery during our project. We observed the eurhythmics only in another class after graduation (see details in S1 Note).

The ages of class units at that time were 5.03 (*SD* = 0.24), 5.28 (*SD* = 0.24), 5.59 (*SD* = 0.25), and 5.71 (*SD* = 0.25) for the five-year-old class in November 2017 and February, May, and July 2018, respectively, and 6.18 (*SD* = 0.27) for the six-year-old class in November 2017. We administered a questionnaire to parents regarding the birth dates of their children. However, we could not obtain the information for one male and three female participants in the five-year-old class and one female participant in the six-year-old class. Additionally, one female participant in the five-year-old class quit during our project. Hence, the average ages were calculated after excluding these children. The *t*-tests indicated that the average age of the six-year-old class was significantly higher than that of the five-year-old class on each measurement date (5.03 age: *t*(19) = 9.726, *p* = .000, Hedges’ *g* = 4.289; 5.28 age: *t*(19) = 7.617, *p* = .000, Hedges’ *g* = 3.359; 5.59 age: *t*(19) = 4.876, *p* = .0004, Hedges’ *g* = 2.150; 5.71 age: *t*(19) = 3.923, *p* = .004, Hedges’ *g* = 1.730) (see details of statistical analysis below procedures in the Analysis section).

The numbers of children participating in the activities were 12 (boys: 6; girls: 6), 13 (boys: 6; girls: 7), 13 (boys: 6; girls: 7), and 11 (boys: 5; girls: 6) for the five-year-old class in November 2017 and February, May, and July 2018, respectively, and 11 (boys: 4; girls: 7) for the six-year-old class in November 2017. We should note that some children in both classes were absent from the activities because of illness.

### Informed consent

A nursery school in Tokyo, Japan, cooperated with our project. We first explained the processes of video recording and data collection to the principal of the nursery and the children’s parents. We also explained that children could participate in eurhythmics without being subjects for data processing and analysis. Verbal consent based on our distributed materials was obtained from the principal of the nursery and parents of participating children. Written informed consent was obtained from the principal just before video recording and data collection. Additionally, an opportunity to reject use of the children’s data was provided to the parents as an opt-out method. Our work was approved by the ethics and safety committees of Kyoto Institute of Technology, the University of Electro-Communications, and Tamagawa University, to which we belonged at that time. This study was carried out following all mandatory regulations. The children’s data were carefully processed so that the individuals could not be identified.

### Studied activities

**Fig 1:**
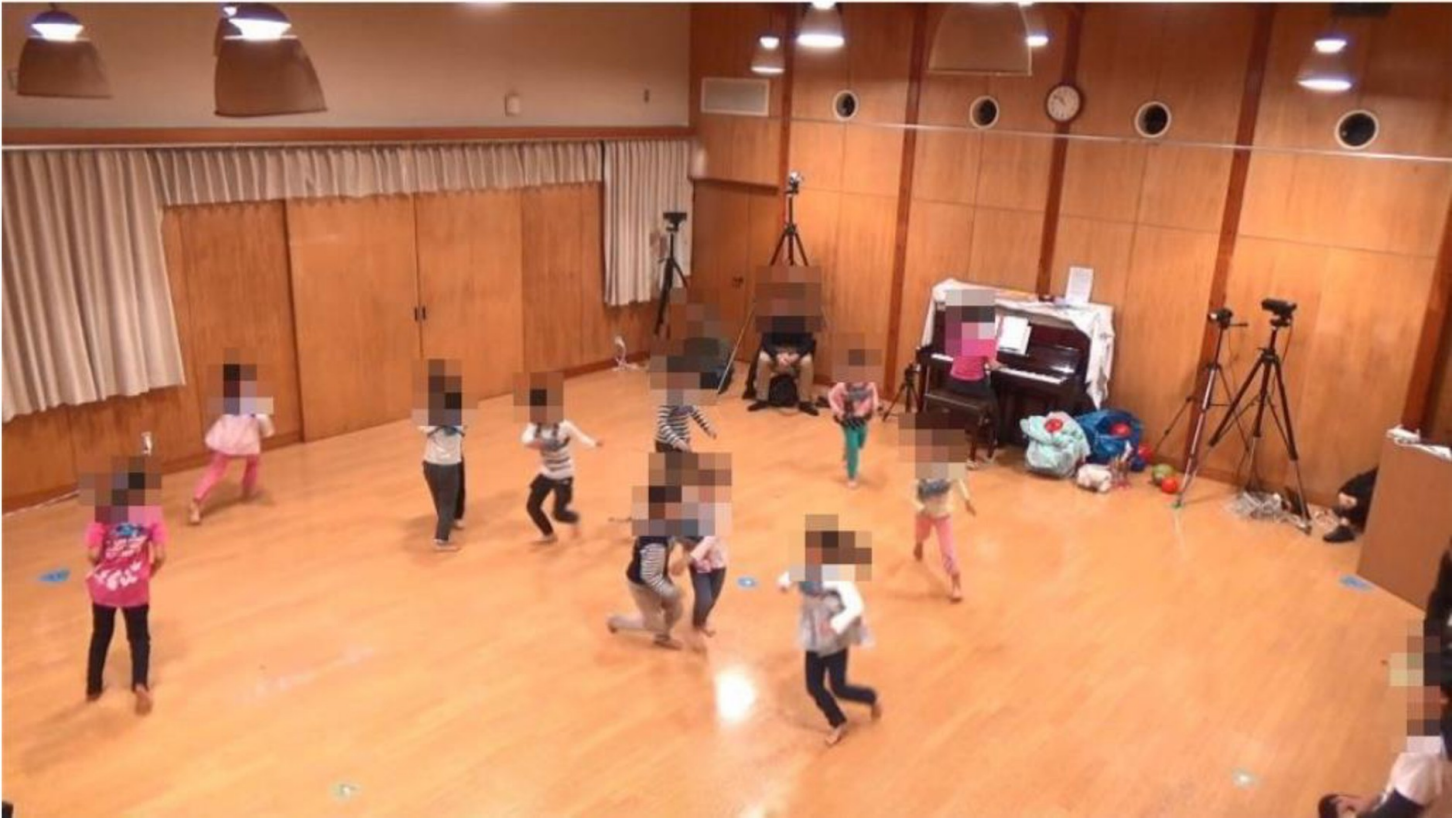
Running activity recorded from a bird’s-eye view, the simplest one in eurhythmics. Children ran freely when the instructor played the piano.

There are various activities involved in eurhythmics for example, throwing a ball and swinging a scarf. This study focused on the running activity, which was the simplest one in eurhythmics. Children ran freely while the instructor played the piano (Fig 1). We modeled the running activity as an object of analysis for the following two reasons. First, it was usually carried out as a warm-up for eurhythmics, although the instructor often changed the overall program on the day according to children’s responses. Second, it had more degrees of freedom compared to other activities because the instructor did not give explicit instructions, such as whom to run with or where to run, unlike other activities in eurhythmics. We therefore expected that there would be many opportunities to observe spontaneous social behaviors while running.

### Video recordings

We recorded eurhythmics in the public hall (Size: 10 m × 8.5 m) near the nursery using a video camera (Sony HDR-CX675) from a bird’s-eye view at about 3 m height (Fig 1) in November 2017 and February, May, and July 2018. The durations for which the instructor played the piano were 61.05, 36.68, 100.59, and 42.18 s for the five-year-old class in November 2017 and February, May, and July 2018, respectively, and 37.11 s for the six-year-old class in November 2017.

## Analysis

### Procedures

The video images (20 fps, 1280 px × 720 px) were recorded from a bird’s-eye view at about 3 m height (Fig 1) and were digitized using motion analysis software to capture the positions of the children in two dimensions (DITECT Co. Ltd., Tokyo, Japan, DIPP-Motion V/2D). Images were digitized manually on a point projected onto the floor from the center of gravity of each child for each four-time frame (0.2 s), and spline interpolation was conducted. When obtaining position data, projective transformation was conducted to correct the distortion caused by the method of recording from a bird’s-eye view. The average of all absolute errors was 0.021 cm × 0.016 cm. All children participated in the activities without wearing reflective clothing to allow them to run freely and comfortably.

**Fig 2:**
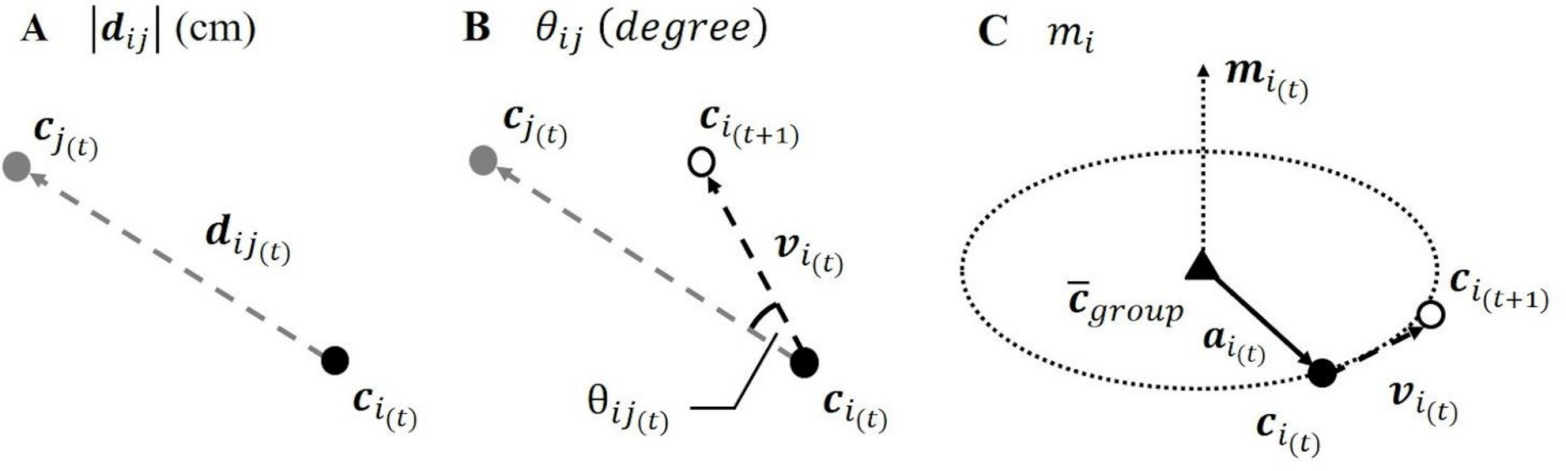
Three indices of children’s group behavior. Illustrated above are $c_{i_{\left(t\right)}}\;and\;c_{j_{\left(t\right)}}$, a pair of children at the current time *t* of the video frame. The time interval is 0.05 s. The first index (A) is the distance |***d***_*j*_| (cm) between a pair of children. The second index (B) is *θ*_*ij*_ (degree), which represents the angle between the velocity vector of a child and the vector composed of positions of a pair of children at the current time, *t*. The linear data ranges from 0 to 180 degrees. If *θ*_*ij*_ is close to 0 degrees, it indicates that a child runs in the direction of another child. The third index (C) is *m*_*i*_, which represents the angular momentum of a child and measures the degree of rotation movement. The range is from 0 to 1. It is calculated by the cross product of the unit vectors composed of the static center of the group of children ${\bar{c}}_{group}$, and the velocity vector of a child.

We analyzed three indices, shown in Fig 2A–2C (see S2 Note to refer to the basic analysis of running activities). They were analyzed in previous studies of group behaviors (e.g., [30,34,48]). The first index (Fig 2A) was the distance |***d***_*ij*_| (cm) between a pair of children. We analyzed the distances between all pairs of children in each time frame. The second index (Fig 2B) was *θ*_*ij*_ (degree), which represents the angle between the velocity vector of a child and the vector composed of positions of a pair of children at the current time, *t*. The linear data ranges from 0 to 180 degrees and included the 0-degree criterion as well as skeletal angle. If the value is close to 0 degrees, it indicates that a child runs in the direction of another child. We analyzed the angles for all pairs of children. The third index (Fig 2C) was *m*_*i*_, which represents the angular momentum of a child. The value ranging from 0 to 1 was calculated using the following equations:

$${c_{group}}_{\left(t\right)}=\frac{1}{N}\sum_{i=1}^{N}{c_{i}}_{\left(t\right)},$$ (1)

$${\bar{c}}_{group}=\frac{1}{T}\sum_{t=1}^{T}{c_{group}}_{\left(t\right)},$$ (2)

$${ua}_{i_{\left(t\right)}}=({c_{i}}_{\left(t\right)}‐{\bar{c}}_{group})/|{c_{i}}_{\left(t\right)}‐{\bar{c}}_{group}|,$$ (3)

$${uv}_{i_{\left(t\right)}}=v_{i_{\left(t\right)}}/|v_{i_{\left(t\right)}}|,$$ (4)

$$m_{i_{\left(t\right)}}=|{ua}_{i_{\left(t\right)}}\times {uv}_{i_{\left(t\right)}}|,$$ (5)

where *i* and *N* represent a child and the number of children, respectively, and *T* represents the number of time frames. ${c_{i}}_{\left(t\right)}$ presents the position of a child at the current time, *t*. Notably, ${c_{group}}_{\left(t\right)}$ was calculated as the position of the children’s group at the current time, *t*. We regarded the mean position through the running activity of all children as the static center of the group of children, ${\bar{c}}_{group}.\;v_{i_{\left(t\right)}}$ represents the velocity vector of a child. The angular momentum of each child, $m_{i_{\left(t\right)}}$, was calculated by the cross product of ${ua}_{i_{\left(t\right)}}$ and ${uv}_{i_{\left(t\right)}}$, the unit vectors defined above. It measures the degree of rotation movement of a child around the static center of the group of children (see S3 Note to refer to the angular momentum of a group unit).

The data of all children for each index were averaged in the normalized frequency of a histogram, which represents the overall characteristics. It was used in a previous study of teamwork in sports science [33]. The purpose of this study was to investigate group behavior around six years of age, linking with the cognition of anticipating others’ behaviors in complex situations based on spontaneous sociality. Therefore, we compared the frequencies of behaviors between the six-year-old class (*M*_*age*_ = 6.18 in November 2017) and the five-year-old class in each age at the measurement date (*M*_*age*_ = 5.03, 5.28, 5.59, and 5.71 in November 2017, February, May, and July 2018, respectively). We should note that one-way ANOVA with the Age factor as a within- or between-subjects factor cannot be conducted in this study. The 6.18 age data were for the six-year-old class, and those of 5.03, 5.28, 5.59, and 5.71 ages were for the five-year-old class; moreover, the numbers of participants differed within the age groups of the five-year-old class. Here, *t*-tests were repeatedly conducted for each bin of a histogram in each index at the 5% level. The *p*-values were corrected by the Bonferroni method to prevent Type I errors and judge significant differences by the Age factor. Thus, only a small amount of data could be acquired in this study. In the future, measurements should be conducted for substantial periods and groups.

If hypothesis #1) is supported, for distance |***d***_*ij*_|, in the bin of the relatively small value, the frequency in the six-year-old class would be higher than that in the five-year-old class. If hypothesis #2 is supported, for angle *θ*_*ij*_, in the bin of around 0 degrees, the frequency in the six-year-old class would be higher. Considering these indices, it can be interpreted that children’s group behavior of the six-year-old class may be more social. Meanwhile, if hypothesis #3 is supported, for angular momentum *m*_*i*_, in the bin of around 1 indicating strong rotation movement, the frequency in the six-year-old class would be lower. It can be indirectly interpreted that the group behavior of the six-year-old class may be more social.

## Results

### Distances between a pair of children

**Fig 3:**
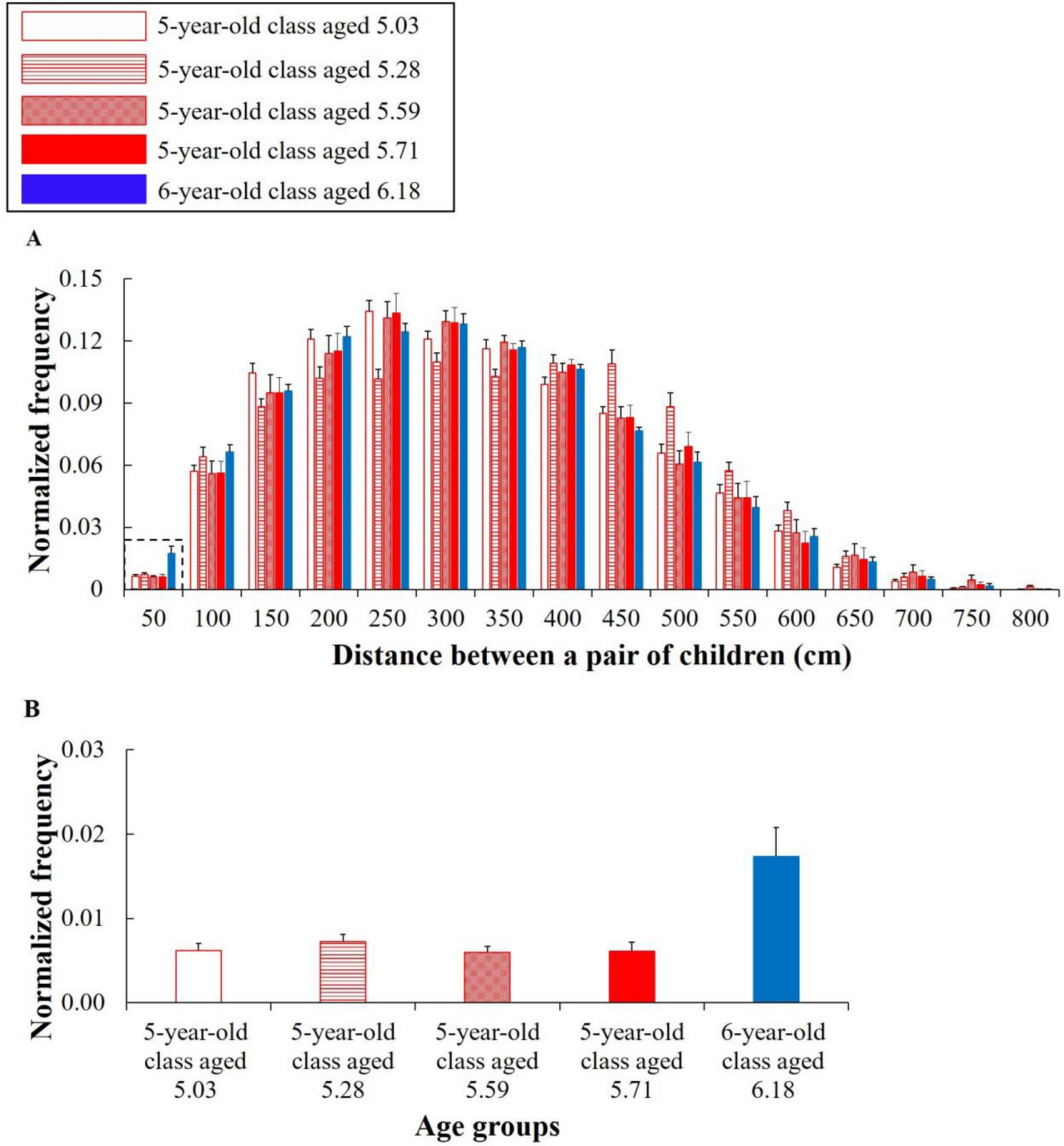
Normalized histograms of the distance |*d*_*ij*_| (cm). The horizontal axes of (A) and (B) represent the bins and age groups, respectively. The vertical axes represent the normalized frequencies. The error bars represent the standard errors. (B) shows the frequencies in the bin of 50 cm. The higher frequency of (B) in the six-year-old class denotes being close enough to touch other children.

For distance |***d***_*ij*_|, there were significant differences between the six- and five-year-old classes in some bins (Fig 3A) (see S4 Note to refer to the detailed results of these statistical measures). Those suggest that the distance between a pair of children was influenced by the Age factor. As a notable result to validate hypothesis #1), the frequency of the six-year-old class (*M*_*age*_ = 6.18) in the bin of 50 cm was significantly higher than that of the five-year-old class at each age (*M*_*age*_ = 5.03, 5.28, 5.59, and 5.71, respectively), and the effect sizes were large (5.03 age: *t*(21) = -3.124, *p* = .021, Hedges’ *g* = 1.304; 5.28 age: *t*(22) = -2.914, *p* = .032, Hedges’ *g* = 1.194; 5.59 age: *t*(22) = -3.333, *p* = .012, Hedges’ *g* = 1.366; 5.71 age: *t*(20) = -2.962, *p* = .031, Hedges’ *g* = 1.263) (Fig 3B). Less than 50 cm is a close distance enough to touch someone. Furthermore, approaching behaviors are represented as positive interpersonal attitudes [27], and the previous studies on the personal space of adults and infants have suggested that 50 cm is the distance at which only those that one feels close to are permitted [25,26]. It can be interpreted that the group behavior in which the distance narrowed to less than 50 cm may include spontaneous social behaviors. This supports hypothesis #1). For a reference outside of that mentioned above, in comparisons with the five-year-old class aged 5.03, 5.59, and 5.71, significant differences were not confirmed for the other bins. In comparison with the five-year-old class aged 5.28, significant differences were confirmed for the bins of 250, 350, 450, and 500 cm (*ps* < .05). (see S4 Note to refer to the detailed results of these statistical measures).

### Angles composed of a pair of children

For angle *θ*_*ij*_, there were not significant differences between the six- and five-year-old classes in all bins (see S4 Note to refer to the detailed results of these statistical measures). To support hypothesis #2, a significant difference by the Age factor was needed in the bin of around 0 degrees. However, similar results were not confirmed at all.

**Fig 4:**
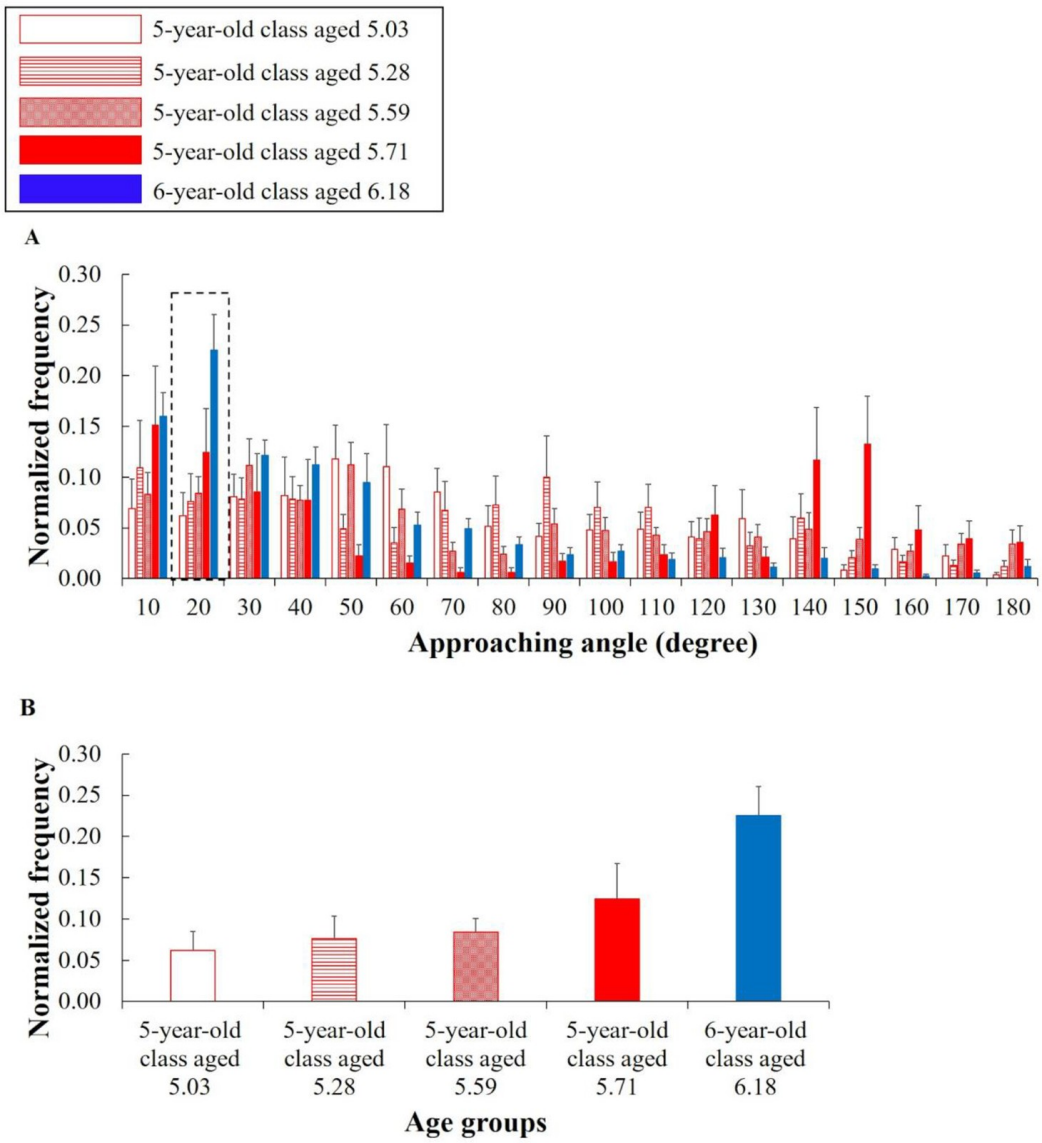
Normalized histograms of the approaching angle *θ*′_*ij*_ (degree) during the period from 0 to 1 s before the approach. The horizontal axes of (A) and (B) represent the bins and age groups, respectively. The vertical axes represent the normalized frequencies. The error bars represent the standard errors. (B) shows the frequencies in the bin of 20 degrees. The higher frequency of (B) in the six-year-old class indicates approaching other children in a short period of time (within one second) to touch them.

Therefore, we performed the following additional analysis. When the distance between the children was less than 50 cm, we analyzed the approaching angle during the periods from 0 to 1 s, from 1 to 2 s, and from 2 to 3 s before the approach. During each period, if the distance was 100 cm or more and less than 200 cm, *θ*_*ij*_ was calculated as the approaching angle *θ*′_*ij*_ (see details in S5 Note). Fig 4A shows the histograms of *θ*′_*ij*_ during the period from 0 to 1 s before the approach. As a notable result, during this period, the frequency of the six-year-old class in the bin of 20 degrees was significantly higher than that of the five-year-old class at each age excluding that of the five-year-old class aged 5.71, and the effect sizes were large (5.03 age: *t*(21) = -3.804, *p* = .004, Hedges’ *g* = 1.588; 5.28 age: *t*(21) = -3.179, *p* = .018, Hedges’ *g* = 1.331; 5.59 age: *t*(21) = -3.559, *p* = .007, Hedges’ *g* = 1.505; 5.71 age: *t*(17) = -1.603, *p* = .509, Hedges’ *g* = .730) (Fig 4B). Hence, this supports hypothesis #2. For a reference outside of that mentioned above, in comparisons with the five-year-old class aged 5.03 and 5.28, significant differences were not confirmed for the other bins. In comparison with the five-year-old class aged 5.59, a significant difference was confirmed for only the bin of 160 degrees (*p* = .012). In comparison with the five-year-old class aged 5.71, a significant difference was confirmed for only the bin of 70 degrees (*p* = .017). During the periods from 1 to 2 s and from 2 to 3 s before the approach, it was considered that these results did not support hypothesis #2 (see S4 Note to refer to the detailed results of these statistical measures).

**Fig 5:**
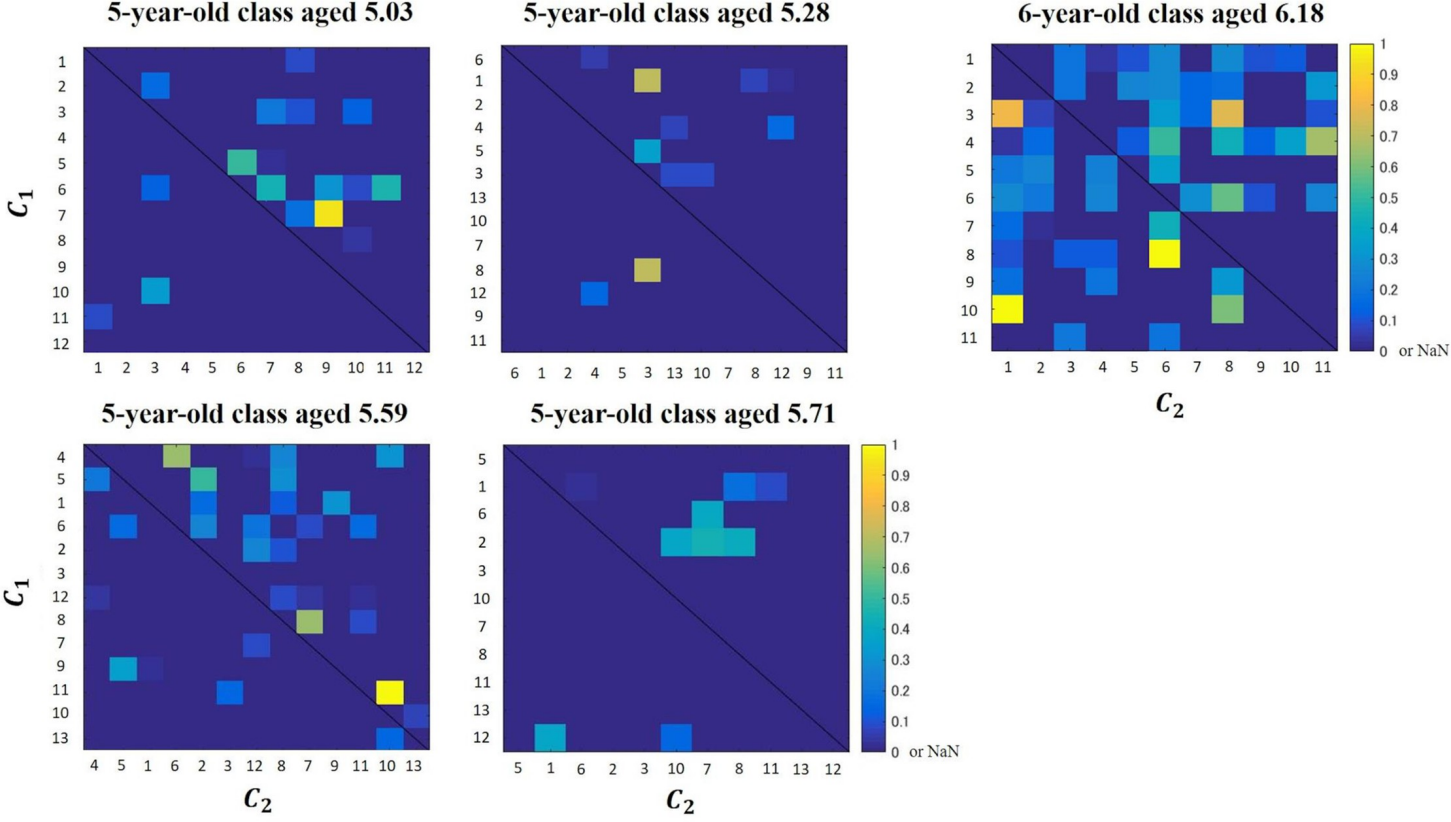
Heat maps of the normalized frequencies of the bin of 20 degrees, at which *c*_1_ approached *c*_2_ during the period from 0 to 1 s before the approach. If a cell has a warmer color, it indicates that the frequency with which *c*_1_ approaches *c*_2_ is higher. For both five- and six-year-old classes, the frequencies of some pairs tend to be higher than those of others (e.g., Pair of children 7 as *c*_1_ and 9 as *c*_2_ of the five-year-old class aged 5.03; Pair of children 8 as *c*_1_ and 6 as *c*_2_ of the six-year-old class). However, the trend of the six-year-old class is weaker than that of the five-year-old class at each age.

Fig 5 shows the heat maps of the normalized frequencies of the bin of 20 degrees, at which *c*_*i*_ approached *c*_*j*_ during the period from 0 to 1 s before the approach. For both five- and six-year-old classes, the frequencies of some pairs tended to be higher than those of others (e.g., Pair of children 7 as *c*_1_ and 9 as *c*_2_ of the five-year-old class aged 5.03; Pair of children 8 as *c*_1_ and 6 as *c*_2_ of the six-year-old class). However, the trend of the six-year-old class was weaker than that of the five-year-old class at each age. Such a social network suggests that universal social behaviors emerged when children were six years old.

### Angular momentum of a child

**Fig 6:**
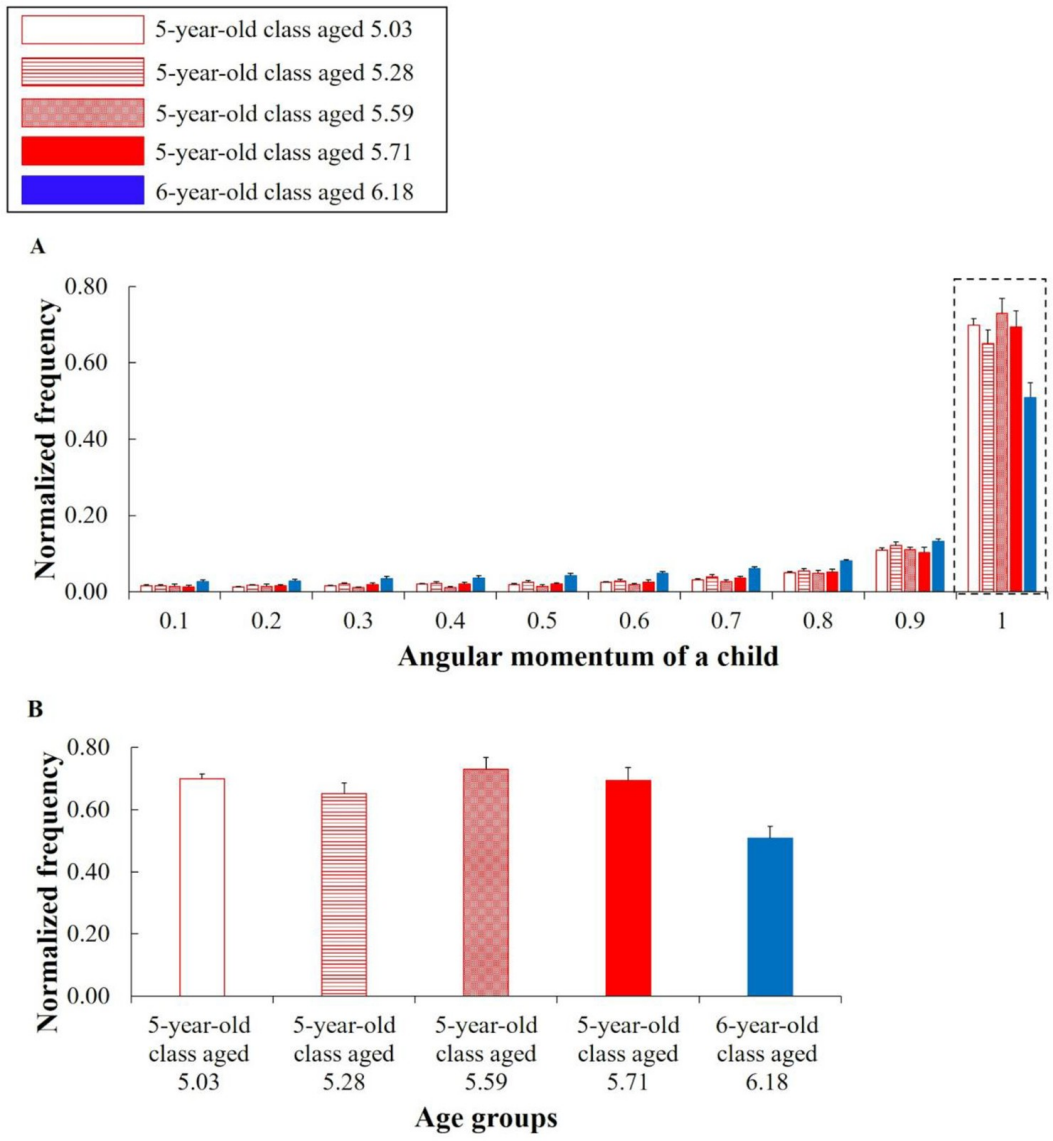
Normalized histograms of the angular momentum of a child *m*_*i*_. The horizontal axes of (A) and (B) represent the bins and age groups, respectively. The vertical axes represent the normalized frequencies. The error bars represent the standard errors. (B) indicates the frequencies in the bin of 1. The higher frequency of (B) in the five-year-old class shows that a larger rotation movement emerges.

For angular momentum *m*_*i*_, there were significant differences between the six-and five-year-old classes in a number of bins (Fig 6A) (see S4 Note to refer to the detailed results of these statistical measures). Those suggest that there were characteristic differences between the age groups in the angular momentum of a child. We focused on the result of the bin of 0.8 and 1 to validate hypothesis #3. In the bin of 0.8, the frequency of the six-year-old class was significantly higher than that of the five-year-old class at each age, and the effect sizes were large (5.03 age: *t*(21) = -5.674, *p* = .000, Hedges’ *g* = 2.368; 5.28 age: *t*(22) = -3.430, *p* = .010, Hedges’ *g* = 1.405; 5.59 age: *t*(22) = -3.494, *p* = .008, Hedges’ *g* = 1.431; 5.71 age: *t*(20) = -2.993, *p* = .029, Hedges’ *g* = 1.276). Meanwhile, in the bin of 1, the frequency of the six-year-old class tended to be significantly lower, and the effect sizes were large (5.03 age: *t*(21) = 4.529, *p* = .001, Hedges’ *g* = 1.891; 5.28 age: *t*(22) = 2.620, *p* = .063, Hedges’ *g* = 1.073; 5.59 age: *t*(22) = 3.916, *p* = .003, Hedges’ *g* = 1.604; 5.71 age: *t*(20) = 3.109, *p* = .022, Hedges’ *g* = 1.326) (Fig 6B). Hence, this supports hypothesis #3. For a reference outside of that mentioned above, in comparison with the five-year-old class aged 5.03 age, significant differences were confirmed for all bins excluding the bin of 0.1 and 0.4 (*ps* < .05). In comparison with the five-year-old class aged 5.28, significant differences were confirmed for the bins of 0.6 and 0.7 (*ps* < .05). In comparison with the five-year-old class aged 5.59, significant differences were confirmed for the bins of 0.3, 0.4, 0.5, 0.6, and 0.7 (*ps* < .01). Additionally, in comparison with the five-year-old class aged 5.71, significant differences were confirmed for the bins of 0.5, 0.6, and 0.7 (*ps* < .05) (see S4 Note to refer to the detailed results of these statistical measures).

## Discussion

### Children’s group behavior

In this study, we introduced a new methodology for quantitative analysis of spontaneous social movement during nursery activity and investigated children’s group behavior using position data. This suggests one of the characteristics of social behaviors in a complex situation. For the distance |***d***_*ij*_| between a pair of children and the approaching angle *θ*′_*ij*_, these results showed that children in the six-year-old class approached other children in a short period of time (within one second) and interacted closely compared to children in the five-year-old class. The instructor did not give explicit instructions, such as whom to run with or where to run. Approaching behaviors represent positive interpersonal attitudes [27]. In addition, the previous studies on personal space have suggested that 50 cm is the distance at which only those that one feels close to are permitted [25,26]. The group behavior in which the distance narrowed to less than 50 cm may, therefore, include spontaneous social behaviors. In the videos, the group behavior of the six-year-old class was like that in a game of tag. Each child approached others in a short period of time (within one second) and those established it.

In the six-year-old class, the normalized frequency in the bin of 50 cm was less than 0.02. The low frequency was based on the characteristic of a game of tag, which requires catching based on the anticipation of the directions in which others escape and escaping based on the anticipation of the directions. In order to establish this social game, such actions would be essential even though the frequency was low. In this study, notable bins are not related to absolute high frequencies, such as in severe traffic accidents. Additionally, if the approaching angle *θ*′_*ij*_ was around 0 degrees, it indicates that a child approached another child. This histogram was composed of 18 bins in 10 degree increments. Hypothesis #2 was supported because the frequency of the six-year-old class in the relatively small value of bin, such as 20 degrees, was higher than those of the five-year-old class. It is important to relatively confirm the values of bins in the histogram, in order to support the hypothesis. However, significant differences by the Age factor for the relatively small values of bins other than 20 degrees were not confirmed and only a small amount of data could be acquired. We should investigate the generalizability of our findings. Furthermore, the duration of running activity was around 37 s. A part of our indices was also used in a previous study in sports science [34]. Group behavior in sports shows characteristic interactions between players over a brief period. Such interactions determine the winners of games within only several seconds. The time of children’s activities in this study was not shorter than that of interactions in sports. Our major standpoint is that children’s group behavior over a brief period would include important information regarding development because childminders in the nursery often estimate children’s various attitudes through experience and momentary observations.

Meanwhile, children in the six-year-old class might play with following implicit rules and norms that were established spontaneously. Such occurrences may be related to the judgment of norms and morals during childhood, and children may develop tendencies to follow group-wide social norms [46,49]. Next, for the angular momentum *m*_*i*_, in the relatively large bin of *m*_*i*_, the frequency of the five-year-old class at each age tended to be significantly higher than that of the six-year-old class. A larger rotation movement around the static center of the group of children emerged in the group behavior of younger children in the five-year-old class.

According to these results, we can interpret this difference in the following way. As one of the developmental processes, the group behavior of the five-year-old class may be related to perceptually-guided anticipation of others’ behaviors based on sociality (e.g., [11–13]) and is relatively primitive. Such organized group behavior is common in many animals, such as rotation movement (e.g., [30,48]). Considering the information processing ability of such fish based on the findings of previous studies (e.g., [30,48]), the anticipation required for rotation movement may be perceptually guided. On the other hand, the group behavior of the six-year-old class may be related to the cognition of anticipating others’ behaviors in more complex situations [9,10] and is relatively social. Humans can recursively estimate various internal states of others, including anticipating others’ behaviors [50]. This would indicate that humans have more advanced cognitive abilities of estimation and anticipation than other animals. Therefore, such anticipation of six-year-old children may be cognitively guided and the related characteristics may not emerge in the group behaviors of fish, birds, or younger children. However, the results only suggest the possibility that the differences in children’s group behavior between age groups might be related to the findings of cognitive development in the previous studies [9,10]. A long-term measurement and a large amount of data would be needed to discuss the generalizability of our findings.

We should note some problems with the above discussions. The limitations of our work are discussed below. Approaching others in this study would also include accidental behaviors not related to the cognition of anticipating others’ behaviors, or aggressive actions. It is difficult for us to distinguish such behaviors through quantitative analysis using the position data of children, which cannot capture their gestures and sharing of intention. Additionally, the emergence of group behavior in the six-year-old class might be accidental because of the differences in children’s personality traits between the classes, the length of living together in the nursery, or the familiarity through the time spent. Such factors other than age might influence children’s group behavior. Meanwhile, it is predicted that, during childhood, playing becomes sociable and may reflect the cognition of anticipating others’ behaviors in complex situations, according to the findings of previous studies related to social interactions of children [22,47] and the development of estimating the internal states of others [9,10]. Following the findings of these previous studies, it is difficult to conclude that our results were based only on the length of living together in the nursery or the familiarity through the time spent. Children’s group behavior might reflect the cognitive development of anticipating others’ behaviors based on spontaneous sociality. The group behavior of the five-year-old class may emerge similarly to that of the six-year-old class (see details in S6 Note to refer to the results of within-subjects analysis in the five-year-old class, using the linear mixed-effects model). Moreover, approaching others observed in this study would include both the behaviors of one child approaching another child (one-sided) and two or more children approaching each other (reciprocal). Children’s group behavior is complex because they do not always move based on rules but often act on impulses during activities. It is difficult to precisely distinguish the two behaviors mentioned above. Lastly, our findings indicated the characteristics of the group behavior demonstrated by the six-year-old children who had established relationships with each other and experienced the naturalistic environment of eurhythmics. If six-year-old children who have not established relationships with each other or have never participated in eurhythmics run freely, we should consider whether these results would be comparable to the results obtained in our work. We need to investigate children’s social interactions in a controlled experimental environment and some conditions based on the findings of this study.

In future studies, some investigations are required to discuss the influence of the cognitive development of anticipating others’ behaviors based on spontaneous sociality to children’s group behavior. We should analyze group behaviors of six-year-old children in other nurseries and compare the results with those of the present study. It is also necessary to record videos periodically for five-year-old children’s activities and investigate the changes in group behavior with increase of age. These works would provide to the generalizability of the characteristics of group behavior of six-year-old children. In addition, we need to analyze spontaneous diffusion and aggregation according to the work of Attanasi et al. [51] and investigate their characteristics related to the cognition of anticipating others’ behaviors in complex situations based on spontaneous sociality and rules of playing. This type of work may be beneficial for developing a more comprehensive understanding of the behaviors of a group unit. Ideal indices to quantitatively analyze complex and random group behavior, which is too difficult to explain characteristics by descriptions through observation, should be developed. Moreover, multi-agent simulation, in which a parameter associated with anticipating others’ behaviors is set, such as in the study by Karamouzas et al. [52] on statistical mechanics, should be utilized to investigate the mechanisms by which the actions of each child influence the group behavior when the value of the parameter changes according to the cognitive development based on the sociality. Such appropriate methodology may help to understand their behaviors, such as distinguishing the actions of one child approaching another child (one-sided) from two or more children approaching each other (reciprocal), as mentioned above. Meanwhile, we need to develop an experimental task, in which researchers can investigate children’s controlled-group behavior to analyze the extent to which the cognition of anticipating others’ behaviors in complex situations develops and the extent to which children follow the rules and norms in the social context.

### Social relationships between children

The heat maps in Fig 5 indicate that, for both five- and six-year-old classes, children tended to approach a few children. However, the trend of the six-year-old class was weaker than those of the five-year-old class. When we showed these heat maps to the instructor of eurhythmics, she reported that the heat maps may reflect the social relationships in the nursery. She also said that children of the six-year-old class played without the bias of communicating with only a few others. Quantitative analysis and visualization based on ecological validity [18,19]; as shown in Fig 5, this would provide meaningful information of social relationships between children and be a means of creating practical and useful curricula and support methods for children in a nursery.

This study proposed a new methodology for the quantitative analysis of spontaneous social movement during nursery activity. Many studies related to sociality have been conducted in developmental psychology and cognitive science; however, it may be difficult to confirm the mechanism of group behavior perceived by the other approaches of controlled experiment and qualitative description because the former sometimes has problems with ecological validity [18,19], and the latter would require too much labor cost for analysis [23,24]. Based on the opinions of previous studies on embodiment and development [27,36–39], we premise that spontaneous sociality is represented by body movement. Fantasia et al. [41] discuss that it is important for understanding sociality and development more deeply to investigate them in a naturalistic environment. This study suggests one of the characteristics of complex interactions in the classroom considering an ecological approach. It provides the findings of embodied sociality in real life that are more interactive than gesture and posture, as discussed by Needham and Libertus [40]. Although our findings have some limitations, they would be meaningful for advancing theories concerning the estimation of the internal states of others, including anticipating others’ behaviors, embodied sociality, and development.

## Conclusion

In this study, we periodically visited the nursery and recorded videos of eurhythmics. This study introduced a new research methodology for the quantitative analysis of spontaneous social movement and investigated children’s group behavior using position data considering an ecological approach. The results showed that children in the six-year-old class approached other children in a short period of time (within one second) and established group behavior like that in a game of tag. It can be interpreted that such interactions between children in the classroom may be more social and related to the cognition of anticipating others’ behaviors in a complex situation. Although only a small amount of data could be acquired, our approach would be meaningful in contributing to advancing theories of developmental psychology and cognitive science (see S7 Note to refer new and original findings from the proceedings in the international conference [45]). Meanwhile, we should note that our work only suggests the possibility that these differences between age groups might be related to the previous findings of cognitive development in developmental psychology and cognitive science. In future work, it is important to record videos periodically and discuss the change in group behavior with increase of age and the generalizability of our findings.

## Supporting information

S1 Note Video recordings. (DOCX)

S2 Note Basic analysis of running activities. (DOCX)

S3 Note Angular momentum of a group unit. (DOCX)

S4 Note Detailed results of the statistical *t*-tests. (DOCX)

S5 Note Algorithm for computing the output of the approaching angle. (DOCX)

S6 Note Detailed results of within-subjects analysis using the linear mixed-effects model. (DOCX)

S7 Note New and original findings from the proceedings in the international conference. (DOCX)

S1 FigTime series $\left|v_{i_{\left(t\right)}}\right|$ (cm/s), which results in the highest and lowest $\bar{v_{i}}$ in the active and non-active children.(PDF)

S2 FigAmounts of running activity $\bar{v_{i}}$ (cm/s).(PDF)

S3 FigAngular momentum of a group unit ${\bar{m}}_{group}$.(PDF)

S4 Fig Running trajectories of the active children. (PDF)

S1 Table Dates of video recordings of running activities. (DOCX)

S2 Table Detailed results of the *t*-tests of the distance |*d*_*ij*_| (cm) between a pair of children. (XLSX)

S3 Table Detailed results of the *t*-tests of angle *θ*_*ij*_ (degree) composed of a pair of children. (XLSX)

S4 Table Detailed results of the *t*-tests of the approaching angle *θ*′_*ij*_ (degree) during the periods from 0 to 1 s, from 1 to 2 s, and from 2 to 3 s before the approach. (XLSX)

S5 Table Detailed results of *t*-tests of the angular momentum *m*_*i*_ of a child. (XLSX)

S6 Table Pseudocode for computing the approaching angle *θ*′_*ij*_ (degree). (DOCX)

S7 Table Detailed results of ANOVAs of the linear mixed-effects model. (XLSX)

S1 Dataset Time series of children’s positions for the five-year-old class aged 5.03. (CSV)

S2 Dataset Time series of children’s positions for the five-year-old class aged 5.28. (CSV)

S3 Dataset Time series of children’s positions for the five-year-old class aged 5.59. (CSV)

S4 Dataset Time series of children’s positions for the five-year-old class aged 5.71. (CSV)

S5 Dataset Time series of children’s positions for the six-year-old class aged 6.18. (CSV)
