# Supplementary material for: Quantitative analysis of spontaneous sociality in children’s group behavior during nursery activity
Source: PLoS One. 2021 Feb 2;16(2):e0246041. doi: 10.1371/journal.pone.0246041 (PMC7853442; doi:10.1371/journal.pone.0246041)
Supplement: S1 Note — (DOCX) [file pone.0246041.s001.docx]

**S1 Note. Video recordings**

This study periodically recorded videos of eurhythmics. There were two types of recording: first, from the side of the hall, and second, from a bird’s-eye view. S1 Table represents the list of dates on which the running activity was conducted. This study analyzed children’s group behavior using position data from the second recordings. Meanwhile, Ichikawa et al. [1] annotated videos on spontaneous social behaviors of each child and specific haptic behaviors were focused on. Such behaviors indicated spontaneous participation in group activities and involvement with other children to establish good relationships. Using the first recordings, these social behaviors in October 2016; January, May, August, and November 2017; and February 2018 were analyzed to hypothesize children’s group behavior around six years of age.

**Reference**

1. Ichikawa J, Fujii K, Nagai T, Omori T, Oka, N. Quantitative analysis and visualization of children’s group behavior from the perspective of development of spontaneity and sociality. In: Rodrigues A, Fonseca B, Preguiça N, editors. Collaboration and Technology: 24th International Conference, CRIWG 2018, Costa de Caparica, Portugal, September 5-7, 2018, Proceedings. Cham: Springer; 2018. pp. 169-176.
