# Supplementary material for: Quantitative analysis of spontaneous sociality in children’s group behavior during nursery activity
Source: PLoS One. 2021 Feb 2;16(2):e0246041. doi: 10.1371/journal.pone.0246041 (PMC7853442; doi:10.1371/journal.pone.0246041)
Supplement: S2 Note — (DOCX) [file pone.0246041.s002.docx]

**S2 Note. Basic analysis of running activities**

We analyzed children’s group behavior to investigate whether there were individual differences in the amounts of running activities and whether children switched between active and non-active states during eurhythmics. This study calculated $\bar{v_{i}}$ (cm/s) using equation (1). $\boldsymbol{v}_{i_{(t\boldsymbol{)}}}$ represents the velocity vector of a child. *T* represents the number of time frames.

$\bar{v_{i}}$ = $\frac{1}{T}\sum_{t=1}^{T} \left| \boldsymbol{v}_{i_{(t\boldsymbol{)}}} \right|$, (1)

This analysis compared the average $\bar{v_{i}}$ between the six-year-old class ($M_{age}=6.18$ in November 2017) and the five-year-old class in each age at the measurement date ($M_{age}$= 5.03, 5.28, 5.59, and 5.71 in November 2017, February, May, and July 2018, respectively). We should note that the 6.18 age data was for the six-year-old class and those for 5.03, 5.28, 5.59, and 5.71 ages were for the five-year-old class; moreover, the numbers of participants differed within the age groups of the five-year-old class. Here, the *t*-tests were repeatedly conducted at the 5% level. The *p*-values were corrected by the Bonferroni method to prevent Type I errors and judge significant differences by the Age factor. Regarding children’s group behavior, a higher average $\bar{v_{i}}$ suggests that children would run during eurhythmics relatively more actively.

S1 Fig shows the time series $\left| \boldsymbol{v}_{i_{\left( t \right)}} \right|$ of the active and non-active children, resulting in the highest and lowest $\bar{v_{i}}$. Although we conducted a qualitative analysis, the results suggest that there were individual differences in both five- and six-year-old classes. Additionally, fluctuations in the time series $\left| \boldsymbol{v}_{i_{\left( t \right)}} \right|$ suggest that children switched between active and non-active states during eurhythmics. S2 Fig shows the amounts of running activities $\bar{v_{i}}$ in the five- and six-year-old classes. The *t*-test confirmed that the value in the six-year-old class was significantly lower than that in the five-year-old class aged 5.03, and the effect size was large (*t*(21) = 3.483, *p* = .009, Hedges’ *g* = 1.454). However, significant differences were not confirmed between the six- and five-year-old classes of other age groups (5.28 age: *t*(22) = 1.192, *p* = .985, Hedges’ *g* = .488; 5.59 age: *t*(22) = 1.854, *p* = .309, Hedges’ *g* = .759; 5.71 age: *t*(20) = 1.075, *p* = 1.180, Hedges’ *g* = .459). Hence, the basic analysis did not notably indicate differences in children’s group behavior between the age groups because the individual differences were too large.

**S1 Fig. Time series** $\left| \boldsymbol{v}_{\boldsymbol{i}_{\left( \boldsymbol{t} \right)}} \right|$ **(cm/s), which results in the highest and lowest** $\bar{\boldsymbol{v}_{\boldsymbol{i}}}$ **in the active and non-active children.** The characteristic in which the time series data are missing halfway indicates that children stopped running when the instructor stopped playing the piano. The active child in the five-year-old class aged 5.03 participated in the activity from the middle. The non-active child in the five-year-old class aged 5.28 age stopped participating in the running activity on the way. The lengths of their time series data therefore differ from those of other children. There are individual differences in both five- and six-year-old classes. Fluctuations in the time series suggest that children switched between active and non-active states during eurhythmics.

**S2 Fig. Amounts of running activity** $\bar{\boldsymbol{v}_{\boldsymbol{i}}}$ **(cm/s).** The horizontal and vertical axes represent the age groups and averages, respectively. The error bars represent the standard errors. Regarding children’s group behavior, a higher average $\bar{v_{i}}$ suggests that children would run during eurhythmics relatively more actively. This index does not notably indicate differences in children’s group behavior between the age groups because the individual differences are too large.
