# Supplementary material for: Quantitative analysis of spontaneous sociality in children’s group behavior during nursery activity
Source: PLoS One. 2021 Feb 2;16(2):e0246041. doi: 10.1371/journal.pone.0246041 (PMC7853442; doi:10.1371/journal.pone.0246041)
Supplement: S3 Note — (DOCX) [file pone.0246041.s003.docx]

**S3 Note. Angular momentum of a group unit**

To validate hypothesis #3, this study also analyzed the angular momentum of a group unit $\bar{m}_{group}$. This was based on the previous studies of group behaviors [1,2]. The value ranged from 0 to 1 and was calculated using equations (1) and (2). If hypothesis #3, which indicates running with broken rotation movement to touch other children, is supported, the angular momentum of a group unit in the six-year-old class would be lower than that in the five-year-old class.

${m_{group}}_{(t)}=\frac{1}{N}\left| \sum_{i=1}^{N} \boldsymbol{ua}_{i_{\left( t \right)}}\times\boldsymbol{uv}_{i_{(t)}} \right|,$ (1)

$\bar{m}_{group}$ = $\frac{1}{T}\sum_{t=1}^{T} {m_{group}}_{(t)},$ (2)

$\boldsymbol{ua}_{i_{\left( t \right)}}$ and $\boldsymbol{uv}_{i_{(t)}}$ are unit vectors composed of the static center of the group of children and the velocity vector of a child (see Fig 2 C in the manuscript). *N* represents the number of children. Therein, *t* and *T* represent the current time and the number of time frames, respectively.

Similar to the angular momentum of a child in the manuscript, this analysis also compared $\bar{m}_{group}$ between the six-year-old class ($M_{age}=6.18$ in November 2017) and the five-year-old class in each age at the measurement date ($M_{age}$= 5.03, 5.28, 5.59, and 5.71 in November 2017, February, May, and July 2018, respectively). The *t*-tests were repeatedly conducted at the 5% level. The *p*-values were corrected by the Bonferroni method.

S3 Fig shows the results. The angular momentum of the six-year-old class was significantly lower than that of the five-year-old class at each age, and the effect sizes were large (5.03 age: *t*(1962) = 39.418, *p* = .000, Hedges’ *g* = 1.836; 5.28 age: *t*(1474) = 37.621, *p* = .000, Hedges’ *g* = 1.959; 5.59 age: *t*(2750) = 65.274, *p* = . 000, Hedges’ *g* = 2.806; 5.71 age: *t*(1586) = 35.648, *p* = . 000, Hedges’ *g* = 1.793). This supports hypothesis #3. It was confirmed that a larger rotation movement emerged in the five-year-old class. The group behavior was as concrete as the milling state of schools of fish [2]. S4 Fig shows the running trajectories of the active children in S1 Fig, related to S2 Note. Although we conducted a qualitative analysis, these results suggest that the trajectories of the five-year-old class were more stable and tended to converge more uniquely than that of the six-year-old class.

**S3 Fig. Angular momentum of a group unit** ${\bar{\boldsymbol{m}}}_{\boldsymbol{group}}$**.** The horizontal and vertical axes represent the age groups and the degree of rotation movement of a group unit, respectively. The minimum and maximum values of $\bar{m}_{group}$ are 0 and 1. The error bars represent the standard errors. It is confirmed that the rotation movement of the five-year-old class is larger and near the milling state.

**S4 Fig. Running trajectories of the active children.** This suggests that the trajectories of the five-year-old class are more stable and tend to converge more uniquely than that of the six-year-old class.

**References**

1. Couzin ID, Krause J, James R, Ruxton GD, Franks NR. Collective memory and spatial sorting in animal groups. J Theor Biol. 2002;218: 1-11.
2. Tunstrøm K, Katz Y, Ioannou CC, Huepe C, Lutz MJ, Couzin, ID. Collective states, multistability and transitional behavior in schooling fish. PLoS Comput Biol. 2013 Feb 28. doi:10.1371/journal.pcbi.1002915
