# Supplementary material for: Quantitative analysis of spontaneous sociality in children’s group behavior during nursery activity
Source: PLoS One. 2021 Feb 2;16(2):e0246041. doi: 10.1371/journal.pone.0246041 (PMC7853442; doi:10.1371/journal.pone.0246041)
Supplement: S4 Note — (DOCX) [file pone.0246041.s004.docx]

**S4 Note. Detailed results of the statistical *t*-tests**

S2, S3, S4, and S5 Tables represent the detailed results of *t*-tests in the manuscript for #1) the distance $\left| \boldsymbol{d}_{ij} \right|$ (cm) between a pair of children; #2) the angle $\theta_{ij}$ (degree) composed of a pair of children; #3) the approaching angle ${\theta^{'}}_{ij}$ (degree) during the periods from 0 to 1 s, from 1 to 2 s, and from 2 to 3 s before the approach; and #4) the angular momentum $m_{i}$ of a child.
