# Supplementary material for: Quantitative analysis of spontaneous sociality in children’s group behavior during nursery activity
Source: PLoS One. 2021 Feb 2;16(2):e0246041. doi: 10.1371/journal.pone.0246041 (PMC7853442; doi:10.1371/journal.pone.0246041)
Supplement: S5 Note — (DOCX) [file pone.0246041.s005.docx]

**S5 Note. Algorithm for computing the output of the approaching angle**

S6 Table represents the algorithm for computing the output of the approaching angle ${\theta^{'}}_{ij}$ in the pseudocode. When the distance between a pair of children was less than 50 cm, this algorithm computed the approaching angle during the periods from 0 to 1 s, from 1 to 2 s, and from 2 to 3 s before the approach. During each period, if the distance was 100 cm or more and less than 200 cm, $\theta_{ij}$ was calculated as the approaching angle ${\theta^{'}}_{ij}$.
