# Supplementary material for: Quantitative analysis of spontaneous sociality in children’s group behavior during nursery activity
Source: PLoS One. 2021 Feb 2;16(2):e0246041. doi: 10.1371/journal.pone.0246041 (PMC7853442; doi:10.1371/journal.pone.0246041)
Supplement: S6 Note — (DOCX) [file pone.0246041.s006.docx]

**S6 Note. Detailed results of within-subjects analysis using the linear mixed-effects model**

For reference to within-subjects analysis, this study investigated the changes in children’s group behavior within the five-year-old class using the linear mixed-effects model. Each frequency of #1) the bin of 50 cm in the distance $\left| \boldsymbol{d}_{ij} \right|$ between a pair of children, #2) the bin of 20 degrees in the approaching angle ${\theta^{'}}_{ij}$ during the period from 0 to 1 s before the approach, and #3) the bin of 1 in the angular momentum $m_{i}$ of a child was regarded as the dependent variable. In these bins, the differences in the group behaviors between the six- and five-year-old classes emerged.

The age groups of the five-year-old class ($M_{age}$= 5.03, 5.28, 5.59, and 5.71, respectively) was set to fixed effect of the independent variables. In addition, we regarded children as random effect of the independent variables. ANOVAs of the linear mixed-effects model were conducted, considering individual differences and data loss in their absences from the activities because of illness at the 5% level. If group behavior become more social around six years of age, this within-subjects analysis would show that the frequencies of #1) and #2) significantly increase with age while the frequency of #3) significantly decreases.

S7 Table represents the results. Overall, the estimates of the fixed effect of age groups were not significant, suggesting that the values were 0. Therefore, the characteristic changes in children’s group behavior with increase of age were not confirmed in the five-year-old class. However, we should note that a small amount of data could be acquired in this study. Additionally, the group behavior may dramatically change when children reach over six years old. These discussions will be included in future work.
