# Supplementary material for: Quantitative analysis of spontaneous sociality in children’s group behavior during nursery activity
Source: PLoS One. 2021 Feb 2;16(2):e0246041. doi: 10.1371/journal.pone.0246041 (PMC7853442; doi:10.1371/journal.pone.0246041)
Supplement: S7 Note — (DOCX) [file pone.0246041.s007.docx]

**S7 Note. New and original findings from the proceedings in the international conference**

We previously reported the progress results of this study in the proceedings of the international conference [1]．For publication in this journal, we carried out additional measurements as well as additional analysis. We recorded eurhythmics in May and July 2018, and obtained position data of children (see details in S1 Note). Additionally, in the proceedings of the international conference, we did not investigate the third index of the angular momentum of a child in the analysis of children’s group behavior (see Fig 2 C in the manuscript). The analysis reported in the Supporting Information was not also conducted at that time.

Through the works mentioned above, we could discuss the characteristics of children’s group behavior and the cognition of anticipating others’ behaviors based on spontaneous sociality more deeply. According to the results of the first and second indices, and the new third index, this study suggests that the group behavior of the five-year-old class would be related to perceptually-guided anticipation of others’ behaviors is relatively primitive. Meanwhile, that of the six-year-old class would be related to the cognition of anticipating others’ behaviors in more complex situations and is relatively social.

In addition, future work has been clarified. This study confirmed that children in the six-year-old class approached other children in a short period of time (within one second). However, it is difficult to precisely distinguish the two behaviors of one child approaching another child (one-sided) and two or more children approaching each other (reciprocal) because the group behavior is complex and children do not always move based on rules but often act on impulses during activities. Thus, we need to consider appropriate methodologies, such as multi-agent simulation, in which a parameter associated with anticipating others’ behaviors is set, as in the study by Karamouzas et al. [2]. This may lead to the discussion of various developmental stages by changing some parameters composing behaviors. Furthermore, we have clarified that it is necessary to develop an experimental task in which researchers can investigate children’s controlled-group behavior to analyze the extent to which the cognition of anticipating others’ behaviors in complex situations develops (see details below children’s group behavior and social relationships between children in the Discussion sections).

Therefore, the main additional works provide new and original findings sufficient to publish this study to the journal.

**References**

1. Ichikawa J, Fujii K, Nagai T, Omori T, Oka, N. Quantitative analysis and visualization of children’s group behavior from the perspective of development of spontaneity and sociality. In: Rodrigues A, Fonseca B, Preguiça N, editors. Collaboration and Technology: 24th International Conference, CRIWG 2018, Costa de Caparica, Portugal, September 5-7, 2018, Proceedings. Cham: Springer; 2018. pp. 169-176.
2. Karamouzas I, Skinner B, Guy SJ. Universal power law governing pedestrian interactions. Phys Rev Lett. 2014 Dec 2. doi: 10.1103/PhysRevLett.113.238701
