# Supplementary figures and images for: Quantitative analysis of spontaneous sociality in children’s group behavior during nursery activity
Source: PLoS One. 2021 Feb 2;16(2):e0246041. doi: 10.1371/journal.pone.0246041 (PMC7853442; doi:10.1371/journal.pone.0246041)

# S1 Fig.

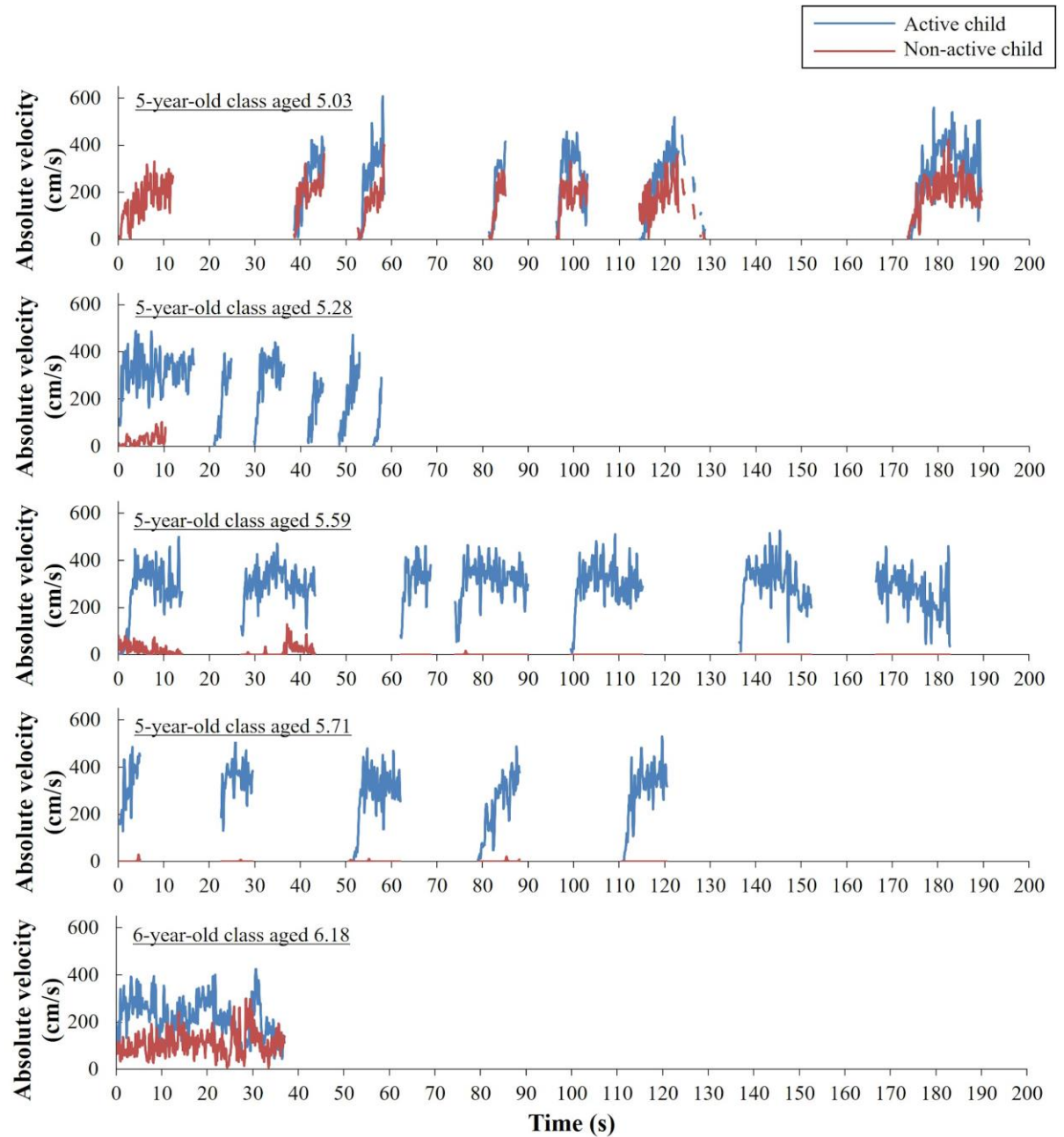

Supplement: S1 Fig — (PDF) [file pone.0246041.s008.pdf]

S2 Fig.

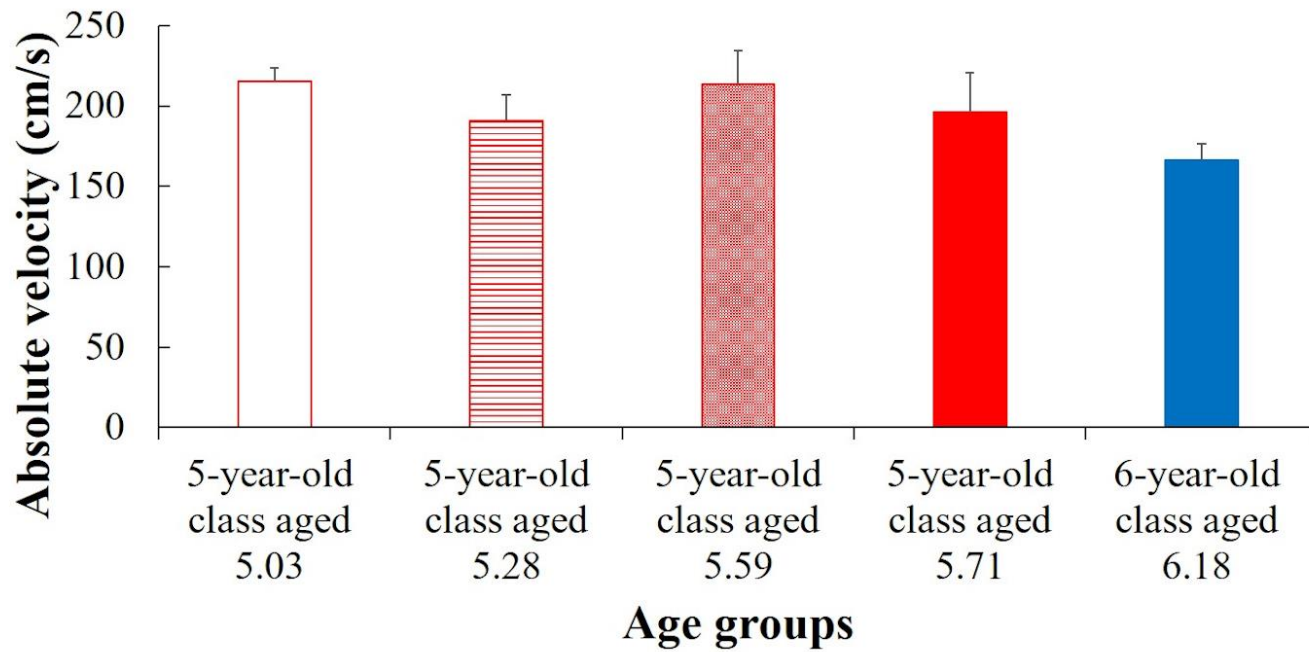

Supplement: S2 Fig — (PDF) [file pone.0246041.s009.pdf]

S3 Fig.

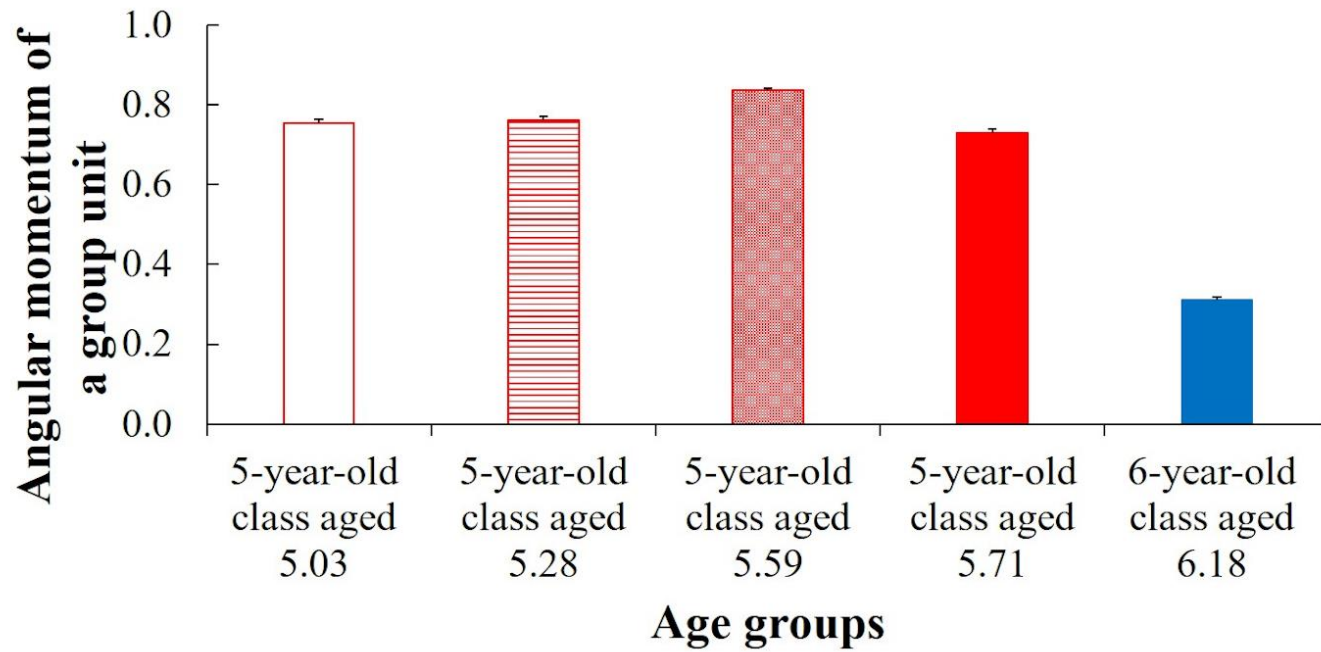

Supplement: S3 Fig — (PDF) [file pone.0246041.s010.pdf]

S4 Fig.

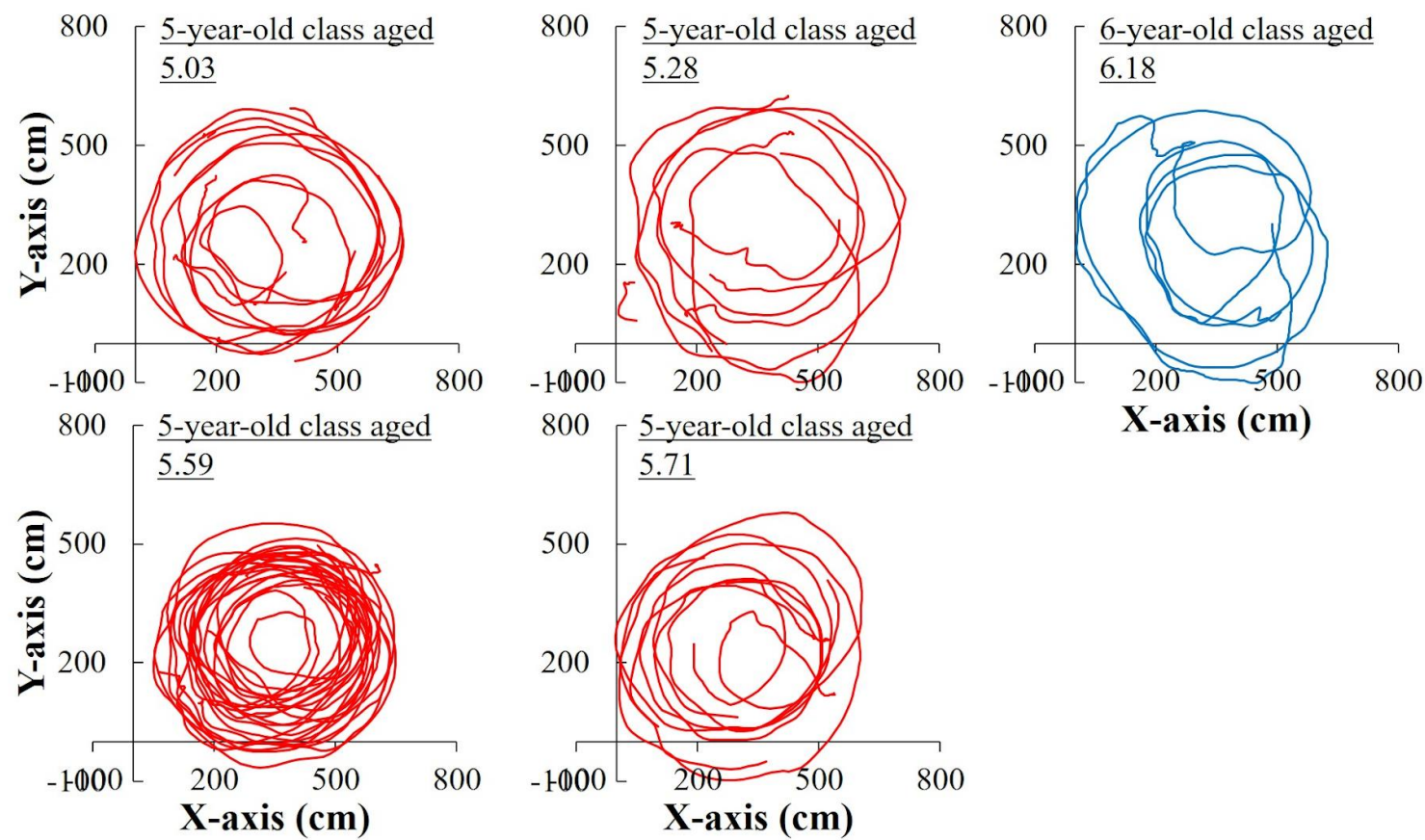

Supplement: S4 Fig — (PDF) [file pone.0246041.s011.pdf]
