# Supplementary material for: Quantitative analysis of spontaneous sociality in children’s group behavior during nursery activity
Source: PLoS One. 2021 Feb 2;16(2):e0246041. doi: 10.1371/journal.pone.0246041 (PMC7853442; doi:10.1371/journal.pone.0246041)
Supplement: S1 Table — (DOCX) [file pone.0246041.s012.docx]

**S1 Table. Dates of video recordings of running activities**

| Date^a^ | Recording from the  side of the hall | | Recording from  a bird’s-eye view | |
| --- | --- | --- | --- | --- |
|  | class A | class B | class A | class B |
| Oct. 2016 | ✔ | ✔ |  |  |
| Jan. 2017 | ✔ | ✔ |  |  |
| May 2017 | ✔ | ✔ |  |  |
| Aug. 2017 | ✔ | ✔ |  |  |
| Nov. 2017 | ✔ | ✔ | ✔ | ✔ |
| Feb. 2018 | ✔ | No activity | ✔ | No activity |
| May 2018 | ✔ | Graduation | ✔ | Graduation |
| July 2018 | ✔ | Graduation | ✔ | Graduation |

^a^At the time recorded from a bird’s-eye view in this study, class A was the five-year-old class and class B was the six-year-old class. We also recorded the running activity of class A in October 2018. However, on that day, the instructor reported that children looked too emotionally unstable to fully engage in eurhythmics. This study excluded the data on that day from all analysis.
