# Supplementary material for: Quantitative analysis of spontaneous sociality in children’s group behavior during nursery activity
Source: PLoS One. 2021 Feb 2;16(2):e0246041. doi: 10.1371/journal.pone.0246041 (PMC7853442; doi:10.1371/journal.pone.0246041)
Supplement: S6 Table — (DOCX) [file pone.0246041.s017.docx]

**S6 Table. Pseudocode for computing the approaching angle** $\boldsymbol{\theta'}_{\boldsymbol{ij}}$ **(degree)**

| Algorithm: Compute output of approaching angle |
| --- |
| Input: $\theta_{{ij}_{(t)}}$  Output: ${\theta^{'}}_{{ij}_{(t)}}$ /* $\theta_{{ij}_{(t)}}$ calculated as the approaching angle */  for 3.0 (sec.) $\leq$ $t_{1}$< *T* (sec.)  for 1 $\leq$ Children *i* $\leq$ *N*  for 1 $\leq\mathrm{Chilren}$ *j* $\leq$ *N*  if 0 (cm) $\leq d_{{ij}_{(t_{1})}} < 50$ (cm)  for $t_{1}$-1.0 (sec.) $\leq$ $t_{2}$ $\leq$ $t_{1}$ (sec.)  if 100 (cm) $\leq d_{{ij}_{(t_{2})}} < 200$ (cm)  ${\theta^{'}}_{{ij}_{(t_{2})}}$ <-- $\theta_{{ij}_{(t_{2})}}$  end  for$t_{1}$-2.0 (sec.) $\leq$ $t_{3}$ $<$ $t_{1}$-1.0 (sec.)  if 100 (cm) $\leq d_{{ij}_{(t_{3})}} < 200$ (cm)  ${\theta^{'}}_{{ij}_{(t_{3})}}$ <-- $\theta_{{ij}_{(t_{3})}}$  end  for $t_{1}$-3.0 (sec.) $\leq$ $t_{4}$ $<t_{1}$-2.0 (sec.)  if 100 (cm) $\leq d_{{ij}_{(t_{4})}} < 200$ (cm)  ${\theta^{'}}_{{ij}_{(t_{4})}}$ <-- $\theta_{{ij}_{(t_{4})}}$  end  end  end  end |
